# Stereotactic Photodynamic Therapy of Recurrent Malignant Gliomas

**DOI:** 10.17691/stm2024.16.2.06

**Published:** 2024-04-27

**Authors:** A.A. Rafaelian, B.V. Martynov, K.A. Chemodakova, A.I. Kholyavin, R.S. Martynov, E.Yu. Klimenkova, M.Yu. Prokudin, G.V. Papayan, I.V. Boykov, D.V. Svistov

**Affiliations:** Neurosurgeon, Neurosurgical Department, Neurosurgery Clinic; Military Medical Academy named after S.M. Kirov, 6 Academician Lebedev St., Saint Petersburg, 194044, Russia; MD, DSc, Associate Professor, Neurosurgeon, Neurosurgical Department, Neurosurgery Clinic; Military Medical Academy named after S.M. Kirov, 6 Academician Lebedev St., Saint Petersburg, 194044, Russia; Neurosurgeon, Neurosurgical Department, Neurosurgery Clinic; Military Medical Academy named after S.M. Kirov, 6 Academician Lebedev St., Saint Petersburg, 194044, Russia; MD, DSc, Neurosurgeon, Neurosurgical Department, Neurosurgery Clinic; Military Medical Academy named after S.M. Kirov, 6 Academician Lebedev St., Saint Petersburg, 194044, Russia; Leading Researcher; Institute of the Human Brain named after N.P. Bekhterevoy, Russian Academy of Sciences, 12A Academician Pavlov St., Saint Petersburg, 197022, Russia; Neurosurgeon, Neurosurgical Department, Neurosurgery Clinic; Military Medical Academy named after S.M. Kirov, 6 Academician Lebedev St., Saint Petersburg, 194044, Russia; Postgraduate Student, Department of Neurosurgery; Military Medical Academy named after S.M. Kirov, 6 Academician Lebedev St., Saint Petersburg, 194044, Russia; PhD, Assistant, Department of Nervous Diseases; Military Medical Academy named after S.M. Kirov, 6 Academician Lebedev St., Saint Petersburg, 194044, Russia; MD, DSc, Senior Researcher, Center for Laser Medicine; Academician I.P. Pavlov First St. Petersburg State Medical University, 6–8 L’va Tolstogo St., Saint Petersburg, 197022, Russia; MD, DSc, Professor, Deputy Head of the Department of Radiology and Radiology; Military Medical Academy named after S.M. Kirov, 6 Academician Lebedev St., Saint Petersburg, 194044, Russia; MD, PhD, Associate Professor, Head of the Department of Neurosurgery; Military Medical Academy named after S.M. Kirov, 6 Academician Lebedev St., Saint Petersburg, 194044, Russia

**Keywords:** malignant gliomas, relapse of glioblastoma, interstitial photodynamic therapy, stereotaxis, glioblastoma, *IDH1* gene mutation

## Abstract

**The aim of the study** is to assess the effectiveness and safety of stereotactic photodynamic therapy (sPDT) with 5-aminolevulinic acid (5-ALA) in patients with recurrent malignant supratentorial gliomas in functionally relevant brain areas.

**Materials and Methods:**

In a retrospective single-center study the results of sPDT with 5-ALA in 10 patients (6 of 10 were male), aged 30 to 62 years (median: 51.5 years; 95% CI: 38–59 years) with recurrent malignant brain gliomas after standard therapy who underwent surgery during the period of 2020–2023 were analyzed. sPDT was conducted during 15 min using 5-ALA at a dosage of 20 mg/kg, a diode laser with a wavelength of 635 nm and power of 1 W, and the LFT-02-BIOSPEC unit (BIOSPEC, Russia). Three patients got repeated sPDT after 3, 7, and 15 months due to a relapse. The number of target points and the optimal position for intervention paths were determined according to the data of preoperative stereotactic MRI of the brain with contrast intensification using the CRW Precision stereotactic navigation system (Integra, USA) and intraoperative registration of the area with the highest intensity of protoporphyrin IX fluorescence along the path (according to fluorescence biospectroscopy).

**Results:**

Glioblastoma (grade IV, WHO) was diagnosed in 7 patients, anaplastic astrocytoma (grade III, WHO) — in 3 persons. Genetic studies were performed for 9 patients, 7 of them had tumors without the *IDH1* gene mutation. None of the patients had a combined 1p/19q deletion. The median volume of the contrast-enhancing part of the recurrent tumor was 7.95 cm^3^ (95% CI: 3.3–13.6 cm^3^). The median time to relapse after sPDT in patients with anaplastic astrocytomas and glioblastomas was 14.5 and 6.5 months, respectively. The median survival time after sPDT in patients with glioblastomas was 15.8 months (95% CI: 0.5–20.1 months), and in patients with anaplastic astrocytomas — 46.3 months (95%, CI not specified). In the early postoperative period, two patients had motor aphasia and hemiparesis, which further regressed.

**Conclusion:**

The results of a small group of patients allow to consider sPDT with 5-ALA as a promising technique to treat patients with recurrent high-grade gliomas in functionally relevant brain areas and require further prospective assessment.

## Introduction

Treatment of patients with relapses of malignant gliomas is a major medical and social issue as there has been no generally accepted treatment strategy specified for them yet. Treatment options include surgery, repeated radiation therapy, and chemotherapy [1, 2].

When functionally relevant brain areas are involved in a relapse, non-surgical treatment techniques are preferable: re-irradiation and/or second-line chemotherapy [3, 4]. At the same time, surgical removal of recurrent tumors helps to increase the overall life expectancy of patients [5].

Surgical treatment of tumors getting into functionally relevant structures may effectively involve various innovative surgical techniques to eradicate tumor cells [6–9]. One them is photodynamic therapy (PDT) [10]. It has been suggested for use in many types of malignant tumors as a local surgical intervention based on cytotoxic effect of a photosensitizing substance that accumulates in the tissue of a malignant neoplasm, including recurrent glioma [11, 12].

Currently, there is a wide enough range of photosensitizers, and the list of such substances continues to grow. These include photofrin II, photoheme, photosens, photoditazine, foscan, temoporfin, hypericin, thalaporfin and 5-aminolevulinic acid (5-ALA) [13, 14]. Unlike other mentioned photosensitizers, 5-ALA is widely used in the routine neuro-oncological practice for intraoperative diagnosis during removal of malignant gliomas. Hence, this medication can also be used for PDT [15].

Several minor studies reported encouraging results of PDT application both during open removal of malignant brain gliomas [16, 17] and during stereotactic surgery on brain tumors [18].

Currently, literature sources describing technical parameters of this technique application are few and heterogeneous, and therefore it seems necessary to study the possibility of using different power and time modes, as well as to assess their effectiveness in stereotactic PDT (sPDT) of recurrent malignant gliomas located in functionally relevant brain areas.

**The aim of the study** is to assess the effectiveness of stereotactic photodynamic therapy with 5-aminolevulinic acid in patients with recurrent malignant supratentorial gliomas in functionally relevant brain areas.

## Materials and Methods

During the period of 2020–2023, a retrospective single-center cross-sectional cohort study was conducted. The data of 10 patients with recurrent malignant brain gliomas, who underwent surgery at the Neurosurgery Clinic of the Military Medical Academy named after S.M. Kirov (Russia) was analyzed; these were 6 men and 4 women aged 30 to 62 years (median: 51.5 years; 95% CI: 38–59 years). Adjuvant therapy was conducted in federal or regional medical institutions. Histological verification of tumors was performed according to the 2016 WHO classification [19]. The study was conducted in accordance with the Declaration of Helsinki (2013) and was approved by the independent Ethics Committee at the Military Medical Academy named after S.M. Kirov. According to the decision of the clinic’s council of doctors, the use of sPDT was considered medically necessary when removal or other types of local stereotactic effects were unsafe or the patient refused them, having the right to new treatment options under trial. All patients submitted their written informed consent for this type of intervention.

### Preoperative preparation

4 h before the intervention, the Alasens aqueous solution of 5-aminolevulinic acid (NIOPIK State Scientific Center, Russia) was administered orally at a dosage of 20 mg/kg, then, the frame of the CRW Precision stereotactic navigation system (Integra, USA) was fixed on the patient’s head under local anesthesia. All patients underwent preoperative contrast-enhanced stereotactic MRI of the brain. Intervention paths and target points in the tumor were simulated in accordance with the results of estimation on a stereotactic phantom by the Integra Radionics software (USA).

### Fluorescence spectroscopy

The surgical intervention was conducted under the combined anesthesia (local administration of the ropivacaine anesthetic 0.5% at a dose of 40 ml and intravenous administration of dexmedetomidine hydrochloride at a dose of 0.8– 1.2 μg/kg/h, followed by dose adjustment after saturation to 0.4–0.8 μg/kg/h). During surgery, the guiding device was aligned along the selected path. The Y-shaped fiber-optic probe of the LESA-01 spectrometer (BIOSPEC, Russia) was put into the brain along the estimated path in the direction of the target point. Along each path, stepby- step, fluorescent biospectroscopy was performed as the probe went each 1 cm deep. Closer to the target point, an increase in fluorescence intensity corresponding to the maximum emission of protoporphyrin IX (PP IX) was recorded. In the area of the highest fluorescence intensity of PP IX along the path and at the target point of the intervention, material was sampled for histological examination.

### Photodynamic therapy

**Figure 1.**
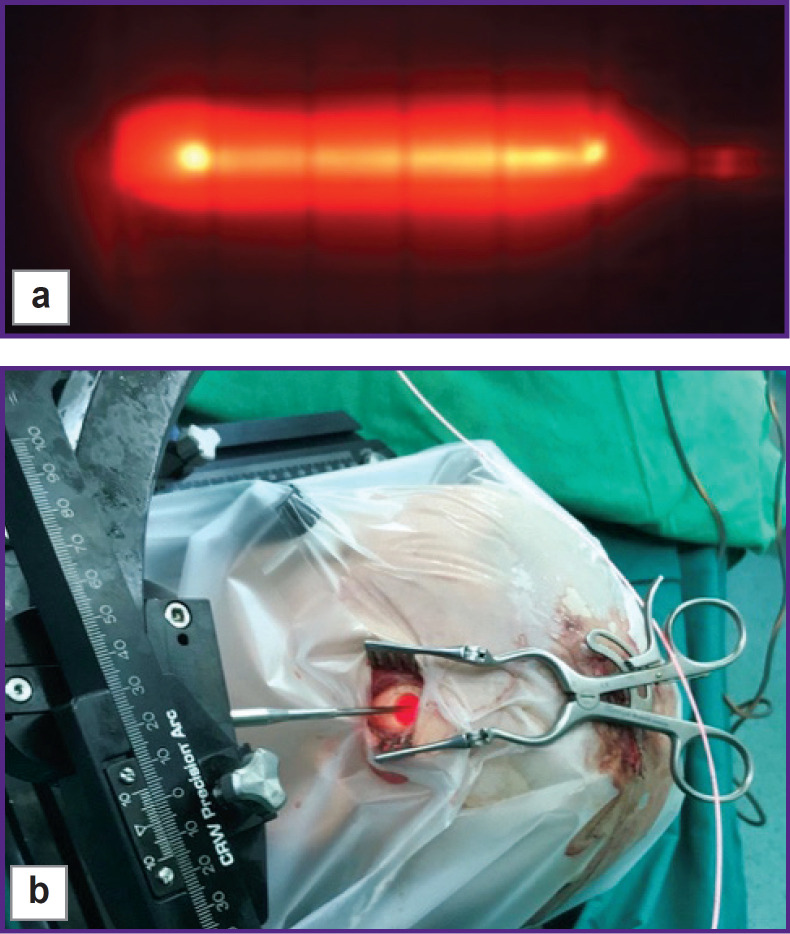
Conduction of stereotactic photodynamic therapy: (a) cylindrical diffuser at the end of a light guide with the laser radiation of 635 nm supplied; (b) intraoperative view during stereotactic photodynamic therapy; the center — the surgical field with the laser enabled

**Figure 2.**
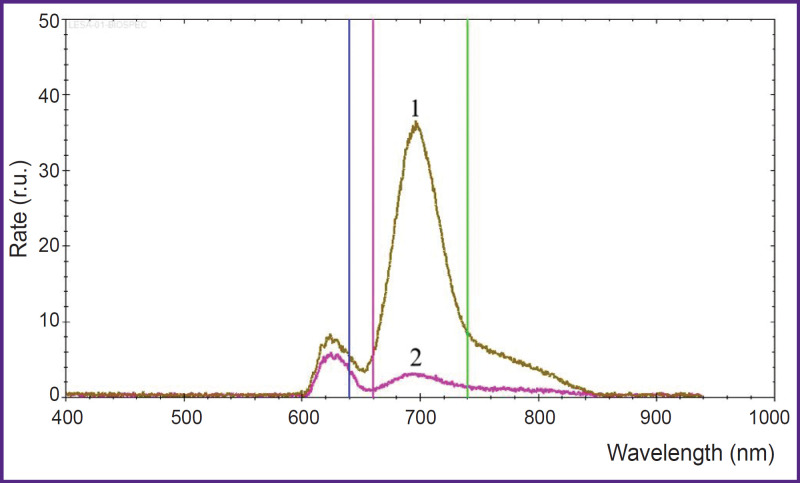
Fluorescence spectrum at the target point of the path: before (*1*) and after (*2*) stereotactic photodynamic therapy, irradiation with a dose of 157 J/cm^2^

After the target point, a light guide with a cylindrical diffuser at the end (diameter — 1.8 mm, length — 22 mm) was inserted into the biopsy cannula (Figure 1) and sPDT was conducted using a diode laser with a wavelength of 635 nm and a power of 1 W for 15 min at each point using the LFT-02-BIOSPEC unit (BIOSPEC, Russia). In each case, the number of target points for sPDT was selected individually in accordance with the estimation based on stereotactic MRI of the brain performed on the day of intervention. When sPDT along all paths was completed, fluorescent biospectroscopy was performed step by step every 1 cm. Here, a significant decrease in the fluorescence intensity of PP IX was seen in the affected areas (Figure 2). During the intervention, neurological status was verbally and visually monitored.

### Postoperative patient management

Postoperative MRI of the brain with intravenous administration of a paramagnetic substance was performed for all patients 24–48 h after sPDT; further, control MRI with intravenous contrast was performed every 2–3 months. The RANO criteria were used to assess if continued growth existed [20].

### Statistical analysis

The analysis of statistical data was conducted using the MedCalc licensed software, v. 10.3.1.0. To objectively assess the effectiveness of sPDT, the authors used the time to tumor development and post-relapse life expectancy from the date of sPDT using the Kaplan–Meier estimate; the log-rank test was used to compare survivorship curves.

## Results

Based on a comparison of data from the earlier study [12] with the results of preoperative planning and postoperative MRI, the authors determined that the phototoxic effect was achieved from the surface of the diffuser to a depth of 4.5 to 6.5 mm (5.5 mm on average), and the laser radiation dose at a given estimated depth was 157±22 J/cm^2^ on average. Energy irradiance was assessed using the QB230 Optical power meter (Advantest Corp, Japan). According to the postoperative MRI study, the approximate dimensions of the alteration after exposure to one diffuser were 3.4×1.3×1.3 cm, whereas the total volume of this area was 2.75±0.23 cm^3^.

The study included 10 patients with verified recurrence of high-grade malignant gliomas in a functionally significant area who underwent sPDT; in three patients, sPDT was repeated after 3, 7 and 15 months due to a relapse.

At the time of sPDT, 7 patients with recurrent tumors had glioblastomas (grade IV, WHO), and 3 patients had anaplastic astrocytomas (grade III, WHO) diagnosed. 2 patients had malignant transformation of the initially diagnosed diffuse astrocytoma of grade II, WHO into glioblastoma and anaplastic astrocytoma during a relapse. Mainly, the tumors were located in the left hemisphere of the brain (7 patients). In 9 people the tumor was localized in functionally significant areas, in 1 it was located in the deep areas of the brain. Clinical symptoms included seizures (6 patients), motor disorders (4 patients), and speech disorders (5 patients). Analysis of molecular and genetic markers was performed in 9 patients. 2 patients had the *IDH1* R132H gene mutation; it was not detected in 7 patients.

*MGMT* gene expression status was assessed in tumors for 9 patients. Expression was low in 7 patients and moderate in 2 patients. A combined 1p/19q deletion was not detected in patients. In 1 patient, analysis of molecular and genetic status was not conducted. The Ki-67 proliferative activity index was determined in 7 patients, the median was 25% (95% CI: 5–45%). The median age at the time of sPDT was 51.5 years (95% CI: 38–59 years), the median volume of the contrasted part of the recurrent tumor was 7.95 cm^3^ (95% CI: 3.3– 13.6 cm^3^). The median Karnofsky score before and after surgery was 90 (95% CI: 80–90) and 85 (95% CI: 70– 90), respectively.

Before sPDT, 7 patients underwent open microsurgical tumor removal, and 3 patients underwent tumor removal using stereotactic cryodestruction. Adjuvant therapy was conducted in federal or regional medical institutions. The median target tumor volume (the volume of the planned area for safe exposure in the contrasted part of the tumor) for sPDT was 6.7 cm^3^ (95% CI: 1.2–12.4 cm^3^), the median number of stereotactic paths was 4 (2–7). At least one irradiation session was performed at each target point along the path. If indicated (residual level of PP IX after irradiation, length of the area of accumulation of the contrast agent), a repeated irradiation session was conducted: either at this target point, or stepped back from it along the same path. The median volume of postoperative changes after sPDT was 4.9 cm^3^ (95% CI: 0.7–8.0 cm^3^). 2 patients had motor aphasia and hemiparesis developed during postoperative period. The neurological deficit was transient and regressed in the early postoperative period.

After intervention, all patients were referred to regional oncology institutions for further treatment. Chemotherapy with temozolomide was conducted in 4 cases, chemotherapy with irinotecan in combination with targeted therapy with bevacizumab — in 6 cases, repeated radiation therapy with chemotherapy and concomitant administration of temozolomide — in 3 cases. Due to tumor progression after combined treatment (sPDT, chemotherapy), 3 patients underwent repeat sPDT.

**Figure 3.**
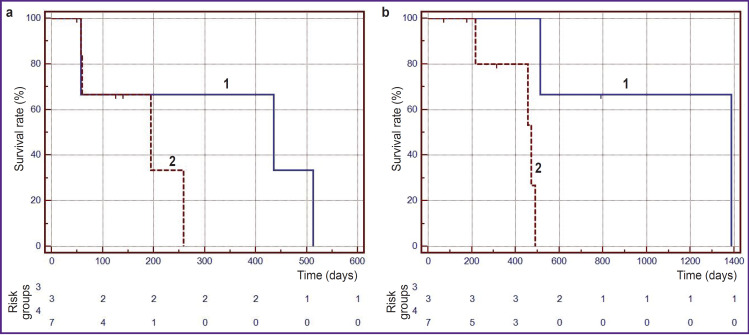
Kaplan–Meier curve: (a) time to disease progression after stereotactic photodynamic therapy for patients with grade III anaplastic astrocytomas (*1*) and grade IV glioblastomas (*2*); (b) life expectancy after stereotactic photodynamic therapy for patients with grade III anaplastic astrocytomas (*1*) and grade IV glioblastomas (*2*)

It was established that the median time to disease progression after sPDT in patients with anaplastic astrocytomas and glioblastomas was 14.5 and 6.5 months, respectively. The 6-month survival-beforeprogression rate in patients with glioblastomas was 33.3% (Figure 3 (a)).

As can be seen on Figure 3 (b) 6- and 12-month survival rates after sPDT in patients with glioblastomas were 80.0 and 53.3%, respectively. The median survival time after sPDT in patients with glioblastomas was 15.8 months (95% CI: 0.5–20.1) months, and in patients with anaplastic astrocytomas — 46.3 months (95%, CI not determined) (p=0.0198, log-rank test). Thus, the histological type of tumor has a significant impact on survival in patients with recurrent malignant gliomas.

**Figure 4.**
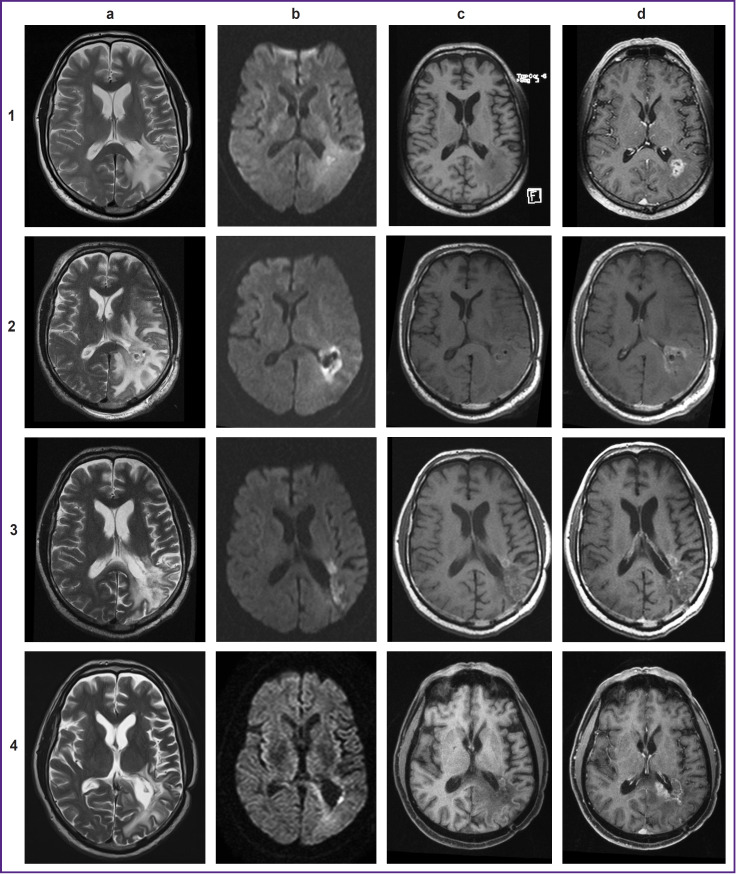
MRI of the brain with contrast intensifying: *1* — before intervention: (a) T2-weighted image (WI) before intervention; (b) diffusion-weighted images (DWI) (b=1000); (c) T1-WI before contrast; (d) post-contrast T1-WIs; *2* — after stereotactic photodynamic therapy: (a) T2-WI, increase in the perifocal area of edema; (b) DWI (b=1000), limitated diffusion in the affected area; (c) T1-WI before contrast, a minor increase in signal intensity in the affected area; (d) T1-WIs post-contrast, decreased accumulation of the contrast agent in the affected area; *3* — 6 months after stereotactic photodynamic therapy: (a) T2-WI, reduction in the area of perifocal edema; (b) DWI (b=1000), limitated diffusion in the affected area and anterior to it; (c) T1-WI before contrast, a minor hyperintense area anterior to the treatment area; (d) post-contrast T1-WIs, a minor area of insignificant accumulation of the contrast agent in the affected area; *4* — 14 months after stereotactic photodynamic therapy (continued growth): (a) T2-WI; (b) DWI (b=1000), remaining minor area of a limitated diffusion in the impact area; (c) T1-WI before contrast; (d) post-contrast T1-WIs, the affected area still has a insignificant accumulation of the contrast agent and a new area of accumulation of the contrast agent measuring 38×36×33 mm

During the postoperative period, patients underwent brain MRI with intravenous contrast every 2–3 months after sPDT. 6 patients had a positive dynamics on MRI: MR signs of a significant decrease in the volume of tumor tissue, a decrease in the accumulation of contrast agent, and the formation of cystic changes in the intervention area. 2 patients lacked area of contrast agent accumulation without a change in tissue volume in the area of exposure, and other 2 patients demonstrated an increase in the area of contrast agent accumulation extending beyond the photodynamic exposure area. In 6 cases, the relapse occurred statistically significantly not in the intervention area, but in a shifted area at a distance of 4.8 mm on average (Figure 4).

**Figure 5.**
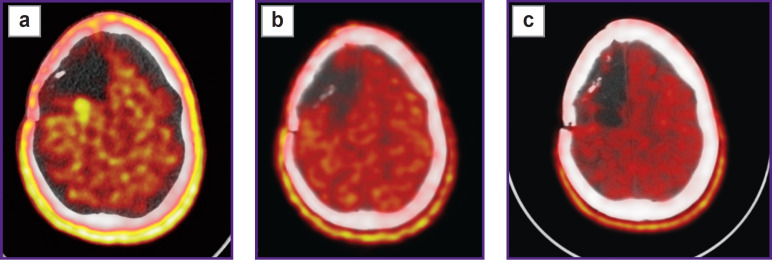
PET/CT with [^11^C] methionine: (a) a radiopharmaceutical agent accumulation area is determined along the posterior contour of the postoperative cyst (accumulation index — 2.4) with the dimensions of 13×24×17 mm; (b) 1 month after stereotactic photodynamic therapy; (c) 9 months after stereotactic photodynamic therapy, no relapse

To differentiate radiation necrosis from continued tumor growth patients underwent PET/CT with [^11^C]-methionine (Figure 5).

## Discussion

Currently, the maximally radical and safe microsurgical removal is the standard treatment for gliomas. Total resection of the primary malignant glioma leads to longer overall survival compared to a partial tumor resection and biopsy, regardless of *IDH* gene mutation status and O6-methylguanine-DNA methyltransferase promoter methylation [21]. There are conflicting reports about significance of surgical resection in treatment of recurrent malignant gliomas [22]. Many patients with recurrent glioblastoma get only conservative treatment, with a median survival of 7–10 months [23, 24]. Only about 20–30% of patients with glioblastomas are candidates for resection of recurrent tumors [25], with the account to the location and volume of tumor that can be safely removed [24].

The location of the tumor in functionally relevant and deep areas of the brain is a significant risk factor for surgery-related complications and the main reason for incomplete removal of glioma [26, 27]. The results of treatment using minimally invasive surgical cytoreduction techniques demonstrated that the said techniques are safe and effective in patients with minor supratentorial gliomas of any location, and, when combined with microsurgical removal, in patients with extensive hemispheric gliomas extending to functionally relevant and deep brain areas [7, 28–30].

The use of photodynamic therapy in treatment of malignant gliomas has become more popular over the last decade, both in open and stereotactic procedures for primary and recurrent tumors [17, 18, 31–33]. This study reports the experience of treating 10 patients with recurrent malignant gliomas located in deep areas or functionally relevant structures of the brain using sPDT; here, patients without the *IDH1*/2 gene mutation predominated (7 out of 9 examined). The median followup period after sPDT was 12 months (2.4–24.4 months).

The treatment results showed that the median time to tumor progression after sPDT was 14.5 and 6.5 months, respectively, in patients with recurrent anaplastic astrocytomas and recurrent glioblastomas, and the median life expectancy was 46.3 and 15.8 months, respectively. These results are comparable with the results of interstitial PDT using 5-ALA in patients with recurrent malignant gliomas reported in the retrospective study that was performed at the University Hospital of Munich [32]. The median time to progression after interstitial PDT in the mentioned study was 6.8 months, and the median post-relapse survivorship time was 12.5 months; here, patients were combined into a unified group of malignant gliomas without specifying the histological type of the recurrent tumor. This does not allow to fully assess the effectiveness of this technique in patients with recurrent gliomas of various histological types. According to the results of the present study, in patients with recurrent malignant gliomas, the histological type of tumor has a significant impact on survival rate (p=0.0198).

In studies [28, 34–36], in patients who underwent microsurgical removal of a recurrent tumor, postrelapse life expectancy ranged from 5 to 13 months, with the maximum increase achieved only with total removal of the contrast-enhancing part of the recurrent glioblastoma. A similar increase in life expectancy (from 9.0 to 11.2 months) after laser interstitial thermotherapy was demonstrated in studies of patients with recurrent glioblastomas [37, 38]. The life expectancy of patients with recurrent glioblastomas after sPDT according to the literature is provided in the Table.

**Table:** Results of stereotactic photodynamic therapy of recurrent malignant gliomas

Author	Number of patients	Tumor volume (cm^3^), Me (min–max)	Grade	Median survivorship rate after stereotaxic photodynamic therapy (months)
Beck, et al. (2007) [12]	10	5.90 (2.10–10.20)	III–IV	15
Johansson, et al. (2013) [39]	5	5.92 (1.50–10.0)	IV	15
Lietke, et al. (2021) [32]	44	3.34 (0.50–22.80)	III–IV	13
Data of the study	10	7.95 (3.20–22.50)	III–IV	Grade IV — 15.8, grade III — 46.3

At the same time, in case of a repeated irradiation or stereotactic radiosurgery, the median post-relapse life expectancy in patients with recurrent unresectable glioblastomas was about 9 months [40], which is significantly lower than the period recorded in this study.

Therefore, the safest surgical cytoreduction (including sPDT) of recurrent malignant gliomas, which are refractory to other treatment techniques, and gliomas, for which microsurgical resection is impossible, increases post-relapse life expectancy, especially in patients with recurrent glioblastomas without an *IDH* gene mutation [38].

## Conclusion

Stereotactic techniques for treatment of recurrent malignant gliomas using various physical factors of interstitial ablation provide an alternative for doctors and patients who are to choose treatment options. The results hereof allow to consider stereotactic photodynamic therapy as a promising technique for treatment of patients with recurrent high-grade gliomas in cases when repeated open surgery has a high risk of neurological deficit development.

Methodology issues related to stereotactic photodynamic therapy, as well as the minimum sufficient and maximum permissible doses of energy for a positive result are still open. To reliably assess the effectiveness of stereotactic photodynamic therapy in treatment of high-grade gliomas, further clinical and experimental interdisciplinary studies are required.
